# The association of *ACTN3* R577X polymorphism with sports specificity in Japanese elite athletes

**DOI:** 10.5114/biolsport.2022.108704

**Published:** 2021-11-10

**Authors:** Nobuhiko Akazawa, Nao Ohiwa, Kazuhiro Shimizu, Natsumi Suzuki, Hiroshi Kumagai, Noriyuki Fuku, Yasuhiro Suzuki

**Affiliations:** 1 Department of Sports Research, Japan Institute of Sports Sciences, Tokyo, Japan; 2 Faculty of International Studies, Takushoku Univeristy, Tokyo, Japan; 3 Graduate School of Health and Sports Science, Juntendo University, Chiba, Japan; 4 Center for General Education, Tokyo Keizai University, Tokyo, Japan

**Keywords:** Elite athletes, ACTN3 R577X polymorphism, Sports events

## Abstract

The α-actinin-3 proteins regulate muscle function and are located in the Z-line of the fast skeletal muscle. A common null polymorphism of R577X in α-actinin-3 gene (*ACTN3*) results in its complete absence in fast-twitch muscles. The *ACTN3* R577X polymorphism is associated with sprint/power performance in athletes. However, little is known about how this polymorphism impacts sports other than sprint/power-oriented sports in Japanese elite athletes. The aim of our study was to examine the association between *ACTN3* R577X polymorphism and elite athlete status in various sports categorized as power/sprint, endurance, artistic, martial arts, and ball game sports. The subjects included 906 Japanese elite athletes and 649 Japanese controls. We analysed the genotype frequency of the *ACTN3* R577X polymorphism in sprint/power (n = 120), endurance (n = 150), artistic (n = 45), martial arts (n = 94), and ball game (n = 497) sports athletes. A higher number of sprint/power athletes were R allele carriers compared to the controls, and the endurance and artistic athletes (OR = 1.69, 1.83, and 2.36, 95% CI: 1.02–2.79, 1.02–3.31, and 1.08–5.13, respectively). The frequency of RR genotype was higher in sprint/power, martial arts, and ball game sports athletes (OR = 1.61, 1.84, and 1.39, 95% CI: 1.04–2.50, 1.11–2.95, and 1.05–1.83, respectively) compared to control. Furthermore, there is a significant linear trend with increasing R allele according to athletic status (P for trend < 0.05). The *ACTN3* R allele is positively associated with sports performance requiring explosive power such as sprint/power, martial arts, and ball game sports categories.

## INTRODUCTION

The sarcometic protein α-actinin-3 is a major component of the Z-line in muscles, which anchors the actin filament in fast-twitch fibres. The α-actinin-3 gene (*ACTN3*) encodes α-actinin-3, and a common null polymorphism in this gene results in the conversion of an arginine (R) residue to a premature stop codon (X) at the amino acid position 577 [1]. The homozygosity of the X allele of the R577X polymorphism in *ACTN3* precludes the expression of α-actinin-3 protein in muscle fibre. Several studies have demonstrated the association of R577X polymorphism with muscle structure and function [2, 3]. Moreover, this polymorphism is associated with the elite athlete status, especially in terms of sprint/power performance [4]. The *ACTN3* R577X polymorphism has been investigated in the context of athletes in power-oriented sports, and RR and RX genotype frequency was found to be higher in European elite sprint/power athletes than in European elite endurance athletes and controls [5, 6]. Likewise, these findings have been replicated in Asian athletes [7, 8]. Although *ACTN3* 577XX genotype may be associated with the performance of European endurance athletes [5], this hypothesis could not be proved in other studies [8–10]. A meta-analysis study has revealed that RR and RX genotypes were associated with athletic performance in sprint/power sports; however, this association could not be confirmed to date because a consensus regarding the association between XX genotype and endurance athletes has not been reached yet [11].

In the Olympic games, other than power- and endurance-oriented sports, in which the results are recorded in centimetres, grams, or seconds (cgs-sports), many sports require mixed power (anaerobic) and endurance (aerobic) fitness; these are categorized into artistic, martial arts, ball game sports, and others [12]. However, artistic sports such as figure skating, martial arts such as judo, and ball game sports such as volleyball and soccer often were not separated from the sprint/power sports requiring anaerobic fitness in previous studies [5, 13]. Most sports disciplines in the Olympic games other than cgs-sports need both aerobic and anaerobic capacity. Though the association of *ACTN3* R577X polymorphism with sprint/power athlete status is widely confirmed, the other categories of sports are more difficult to be investigated due to the different physiological qualities important for performance. Previous studies did not find any significant associations between this polymorphism and athletes status of other sports categories [14, 15]. On the other hand, Heffernan et al. demonstrated that R577X genotype frequency in rugby athletes was different between position-specific characteristics (forwards and backs) [16]. Regarding artistic sports, not only muscular strength and power, but also flexibility and neuromotor coordination are needed to achieve the best performance [17]. Interestingly, the *ACTN3* R577X genotype was associated with flexibility and muscle stiffness in Japanese cohort study [18, 19]. Furthermore, martial arts and ball game sports also rely on explosive and repetitive power generation [20, 21]. In this regard, the duration of an event in martial arts is relatively shorter compared to that in ball game sports (several minutes in martial arts vs. 1–2 hours in ball game sports). An animal study reported that α-actinine-3 knockout mice exhibited greater aerobic enzyme activity and faster recovery from fatigue after exerting force [22]. However, the effect of *ACTN3* R577X polymorphism in artistic or martial arts athletes with sprint/power and endurance athletes has not been investigated separately. Thus, the association of this polymorphism in athletes belonging to such sports categories, especially those that require other fitness parameters such as flexibility or high and middle power capacity, remains unclear.

Therefore, in the present study, we aimed to investigate the association between *ACTN3* R577X polymorphism and sports-specific performance-related characteristics for comparing various sports through a large cohort study. In this study, we first categorized each sports discipline and compared the genotype frequency of R577X polymorphism among these categories. Then, we sub-categorized and analysed the athletes according to their athlete status in these sports categories using a Japanese elite athlete cohort.

## MATERIALS AND METHODS

### Study participants

This study included 906 Japanese athlete (505 men and 401 women) and 649 healthy controls (183 men and 466 women). All the athletes were international-level elite athletes belonging to the national team in their respective sports disciplines and had competed in the Olympics, the World championship, or the Asian games held for their disciplines. Each sports discipline was classified into four categories according to their performance characteristics based on a previous study: cgs-sports, artistic sports, martial arts sports, and ball game sports [12]. Moreover, cgs-sports were divided into sprint/power and endurance sports, based on the predominant energy metabolism system [23]. The subjects were classified based on the sports categories into cgs-sports including sprint/power sports (n = 120) and endurance sports (n = 150), artistic sports (n = 45), martial arts sports (n = 94), and ball game sports (n = 497). The sprint/power sports included track and field sprinters (≤ 400m; n = 45), track and field jumpers (n = 19), track and field throwers (n = 18), bicycle sprinters (n = 7), canoe short distance sprinter (n = 1), short-distance swimmers (≤ 200 m; n = 6), and weightlifters (n = 24). The endurance sports included track and field middle- and long-distance runners (> 800 m) and racewalking athletes (n = 55), middle- and long-distance bicycle track, road, mountain, and pursuit cyclists (n = 23), middle- and long-distance canoe sprinters and slalom athletes (n = 18), middle- and long-distance swimmers (n = 22), triathlon athletes (n = 18), and rowers (n = 14). The artistic sports included artistic gymnasts (n = 14), rhythmic gymnasts (n = 6), trampoline athletes (n = 4), artistic swimmers (n = 7), platform divers (n = 3), and figure skaters (n = 11). The martial arts sports included athletes participating in judo (n = 25), wrestling (n = 15), boxing (n = 7), fencing (n = 29), taekwondo (n = 10), and karate (n = 8). The ball game sports events included badminton (n = 47), baseball (n = 42), basketball (n = 61), volleyball and beach volleyball (n = 75), hockey (n = 52), handball (n = 46), soccer (n = 52), softball (n = 26), tennis (n = 21), table tennis (n = 8), rugby (n = 39), and water polo (n = 28) players. These athletes were recruited newly between 2017 and 2019 at the Japan Institute of Sports Sciences, whilst the controls group included the same subjects included in a previous Japanese study [7]. All participants were informed of the purpose and methods of the study, and each of them provided written informed consent for participation. The study was approved by the institutional ethical committee of the Japan Institute of Sports Sciences and conducted in accordance with the ethical standards of the Helsinki Declaration.

### Genotyping

Genomic DNA was extracted and isolated from venous whole blood through automated nucleic acid extraction using a genomic DNA purification kit (Maxwell RSC, Promega Co., Ltd., Madison, WI, USA) according to the manufacturer’s instructions. Genomic DNA was quantified using a spectrophotometer (e-spect; Malcom Co., LTD., Tokyo, Japan). The *ACTN3* R577X (rs1815739) polymorphism was genotyped by performing TaqMan^®^ SNP genotyping assays (assay ID: C__590093_1_) using a real-time thermal cycler dice (TP850; Takara Bio Inc., Shiga, Japan). First, 10 μL of genotyping mixture containing 5 μL of GTXpress master mix, 0.125 μL of assay mix, and 2.875 μL of distilled water mixed with 2 μL of genomic DNA (1 ng/μL) was used in each reaction. One negative control was included in each plate. Thermal cycling conditions involved an initial denaturation at 95°C for 20 s, followed by 40 cycles of denaturation at 95°C for 3 s, and annealing/extension at 60°C for 20 s. TaqMan assays for genotype calls were analysed using the device software (TP850; Takara Bio Inc., Shiga, Japan).

### Statistical analysis

Chi-squared test was used to detect the presence of Hardy-Weinberg equilibrium (HWE). Odds ratios (OR) and 95% confidence intervals (CI) were calculated to estimate the degree of the contribution of the *ACTN3* R577X genotype to each sport category. The linear-by-linear regression test was used to determine the relation between the *ACTN3* R577X genotype frequency and athlete status of medal acquisition at the Olympic games or at the World Championship (medallists vs. international-level athletes vs. control subjects). Statistical data analyses were performed using SPSS software (version 24; IBM, Armonk, NY, USA) and the statistical significance was set a priori at *P* < 0.05.

## RESULTS

**FIG. 1:**
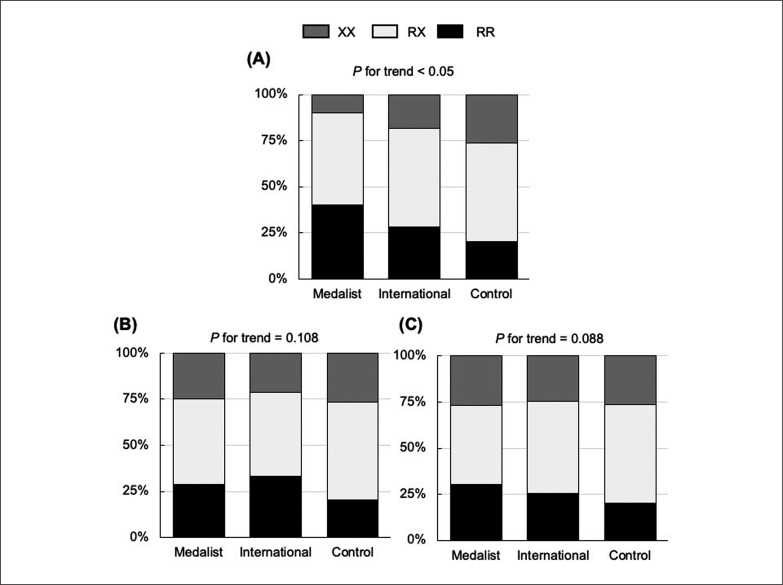
Frequencies of the *ACTN3* genotype in (A) sprint/power athlete, (B) martial arts athlete, and (C) ball game sports athlete dividing competition level.

Table 1 shows the distribution of genotype frequency in the all categories together and each category of sports. The polymorphism distribution in all athletes was in HWE (*P* = 0.986). The OR and 95% CI between each sport category are shown in Table 2. The sprint/power athletes exhibited a higher frequency of the RR + RX genotype than the controls and endurance and artistic athletes exhibited (OR = 1.686, 1.833, and 2.357, respectively; 95% CI = 1.021–2.278, 1.016–3.309, and 1.082–5.134, respectively, *P* < 0.05). The sprint/power, martial arts, and ball game sports athletes had a higher frequency of RR genotype than the control subjects did (OR = 1.613, 1.836, and 1.387, respectively; 95% CI = 1.402–2.498, 1.113–2.949, and 1.052–1.830, respectively, *P* < 0.05). Furthermore, the frequency of RR genotype was different between endurance and martial arts athletes (OR = 0.533, 95% CI = 0.296–0.962, *P* < 0.05). Considering the significant differences between the athletes and the controls demonstrated through odds ratio analyses, we performed a subgroup analysis of the athletic status for medallists (who won Olympic or World championship medals), international-level athletes, and control subjects. There was a significant linear correlation between the frequency of the *ACTN3* RR genotype and the the athletic status in sprint/power sports athletes (*P* for trend < 0.05) (Fig. 1). However, these correlations were not evident in martial arts and ball game sports athletes (*P* for trend = 0.108 and 0.088, respectively).

**TABLE 1:** Genotype frequency of ACTN3 according to sports events and level of competition. n (%)

		All				International				Medalist			

		n	RR	RX	XX	n	RR	RX	XX	n	RR	RX	XX
Total		906	233 (26)	450 (50)	223 (25)	775	193 (25)	387 (50)	195 (25)	131	40 (31)	63 (48)	28 (21)
Athletes	Power	120	35 (29)	64 (53)	21 (18)	110	31 (28)	59 (54)	20 (18)	10	4 (40)	5 (50)	1 (10)
	Endurance	150	30 (20)	78 (52)	42 (28)	128	25 (20)	70 (55)	33 (26)	22	5 (23)	8 (36)	9 (41)
	Artistic	45	8 (18)	22 (49)	15 (33)	27	4 (15)	12 (44)	11 (41)	18	4 (22)	10 (56)	4 (22)
	Martial arts	94	30 (32)	43 (46)	21 (22)	66	22 (33)	30 (46)	14 (21)	28	8 (29)	13 (46)	7 (25)
	Ball game	497	130 (26)	243 (49)	124 (25)	444	111 (25)	216 (49)	117 (26)	53	19 (36)	27 (51)	7 (13)
Control		649	132 (20)	346 (53)	171 (26)								

**TABLE 2:** Odds ratio of genotype for athlete and control subjects according to sports events.

OR (95% CI)								

Sports	XX	RR + RX			XX + RX	RR		
Power								
vs. Control	1.00	**1.686**	**(1.021–2.787)**	*	1.00	**1.613**	**(1.041–2.498)**	*
vs. Endurance	1.00	**1.833**	**(1.016–3.309)**	*	1.00	1.647	(0.940–2.887)	
vs. Artistic	1.00	**2.357**	**(1.082–5.134)**	*	1.00	1.904	(0.806–4.449)	
vs. Martial arts	1.00	1.356	(0.690–2.677)		1.00	0.878	(0.489–1.578)	
vs. Ball game	1.00	1.567	(0.938–2.617)		1.00	1.162	(0.747–1.808)	

Endurance								
vs. Control	1.00	0.920	(0.619–1.368)		1.00	0.979	(0.628–1.526)	
vs. Power	1.00	**0.740**	**(0.302–0.985)**	*	1.00	0.607	(0.346–1.064)	
vs. Artistic	1.00	0.815	(0.463–1.435)		1.00	1.156	(0.488–2.739)	
vs. Martial arts	1.00	0.855	(0.405–1.351)		1.00	**0.533**	**(0.296–0.962)**	*
vs. Ball game	1.00	1.286	(0.567–2.628)		1.00	0.706	(0.451–1.104)	

Artistic								
vs. Control	1.00	0.715	(0.376–1.362)		1.00	0.847	(0.385–1.862)	
vs. Power	1.00	**0.424**	**(0.195–0.924)**	*	1.00	0.525	(0.222–1.240)	
vs. Endurance	1.00	0.778	(0.380–1.590)		1.00	0.855	(0.365–2.049)	
vs. Martial arts	1.00	0.575	(0.262–1.264)		1.00	0.461	(0.192–1.111)	
vs. Ball game	1.00	0.665	(0.346–1.276)		1.00	0.610	(0.277–1.245)	

Martial arts								
vs. Control	1.00	1.124	(0.742–2.083)		1.00	**1.836**	**(1.113–2.949)**	*
vs. Power	1.00	0.735	(0.375–1.450)		1.00	1.138	(0.634–2.045)	
vs. Endurance	1.00	1.352	(0.740–2.469)		1.00	**1.875**	**(1.039–3.383)**	*
vs. Artistic	1.00	1.738	(0.791–3.819)		1.00	2.168	(0.900–5.220)	

vs. Ball game	1.00	1.156	(0.683–1.956)		1.00	1.323	(0.821–2.113)	
Ball game								
vs. Control	1.00	1.076	(0.823–1.407)		1.00	**1.387**	**(1.052–1.830)**	*
vs. Power	1.00	0.638	(0.382–1.066)		1.00	0.86	(0.553–1.338)	
vs. Endurance	1.00	1.170	(0.776–1.763)		1.00	1.417	(0.906–2.216)	
vs. Artistic	1.00	1.504	(0.783–2.888)		1.00	1.638	(0.744–3.610)	
vs. Martial arts	1.00	0.865	(0.511–1.464)		1.00	0.756	(0.469–1.218)	

Bold and * indicates statistical significance.

## DISCUSSION

In the present study, we first investigated the association of *ACTN3* R577X polymorphism with multiple sports categories in Japanese elite athletes. The results demonstrated that the genotype frequency of the R allele in sprint/power sports athletes was higher than in endurance and artistic sports athletes and non-athlete controls, consistent with the results of previous studies [5, 8, 17]. Furthermore, we found that the 577RR genotype frequency was higher in sprint/power, martial arts, and ball game sports athletes compared to controls, and showed a linear trend corresponding to the elite athletic status in sprint/power sports. These results suggest that the number of R alleles in the *ACTN3* R577X polymorphism is associated with sports requiring higher muscular strength.

Sprint/power, martial arts, and ball game sports require explosive force generated at a high speed and powerful and repetitive movements. Previous studies have examined sprint/power sport athletes as well as ball game sports (soccer and volleyball) and martial arts (judo) athletes [5, 13]. We found that the frequency of RR genotypes compared to the XX and RX genotypes in ball game sports and martial arts athletes were higher than in the control subjects. Santiago et al. reported that professional soccer athletes including international and national level players in Spanish club teams had a higher frequency of RR genotype than did control subjects [24]. However, the precise physiological differences between homozygous and heterozygous R allele carriers (RR vs. RX genotype) was not fully understood. In a study on non-athletic control subjects, healthy adolescents with RR genotype were faster at sprinting than those with RX or XX genotypes [25]. Recently, Hogarth et al. compared the effect of *ACTN3* R allele dose on wild-type (W), heterozygous (H), and knock-out (KO) phenotype mice and found that R577X polymorphism was associated with α-actinin-3 mRNA and protein in a dose-dependent manner [26]. It is possible that the RR genotype may play a critical role in conferring the ability to perform at the international level in anaerobic metabolism-related sports including sprint/power sports as well as martial arts and ball game sports.

The current study revealed a linear trend involving the *ACTN3* R577X polymorphism and athletic performance in sprint/power sports athletes. This result was corroborated by a Japanese cohort study which reported the association of *ACTN3* R577X polymorphism with athletic performance level whether it was regional, national, or international in power sports athletes [8]. Furthermore, Mikami et al. reported that sprinters carrying the R allele had the best sprint times for the 100 m sprint than those with the XX genotype, and no sprinter with the XX genotype qualified for the Olympics [7]. We confirmed our supposition regarding higher levels of athletic performance, namely, winning medals was associated with the frequency of *ACTN3* R577X polymorphism in power sport athletes. In contrast, there was no such association in martial arts and ball game sports athletes. Both martial arts and ball game sports require not only the repetition of powerful and explosive action but also endurance and continuous exertion, thereby utilizing both anaerobic and aerobic energy [27]. These results imply a stronger association between *ACTN3* R577X genotype in power-oriented sports athletes and higher level of athletic performance than in athletes of other sports categories.

In this study, there were differences in the distribution of R577X polymorphism between sprint/power athletes and endurance and artistic athletes. A previous study has reported that *ACTN3* R577X polymorphism distribution and allelic frequency were significantly different between sprinters and endurance runners [28]. In this regard, our results showed no association of the R577X polymorphism between endurance athletes and controls even with large sample sizes involving athletes with a high level of performance. This suggests that R577X polymorphism is unlikely to be related to endurance performance. However, the performance of artistic sports athletes including gymnasts also depends mainly on the high- power anaerobic component and flexibility [29]. Elite artistic gymnasts have a lower frequency of XX genotype compared to the control subjects [30]. Conversely, another study has demonstrated that artistic gymnastic athletes with XX genotype exhibited higher training volume and performance quality than RX + RR genotype athletes [31]. These studies discussed that the artistic athletes with XX genotype may have superior neuromotor coordination or trainability in terms of anaerobic capacity [31]. In this study, we found a lower RX + RR genotype frequency in the artistic sports athletes compared to sprint/power athletes, whereas there was no association with athletic status. These discrepancies among stuides may be due to the differences in sample size and/or ethnic group. Moreover, a Japanese cohort study reported a difference in the trunk flexibility between individuals with XX genotype and those with RX + RR genotype [18]. The present study was conducted in larger cohorts which included rhythmic gymnasts, trampoline athletes, artistic swimmers, platform divers, and figure skaters as well as artistic gymnasts. Taken together, the *ACTN3* 577RR genotype seems to be inversely associated with endurance and artistic sports that require aerobic fitness and flexibility fitness compared to sprint/power sports that require anaerobic fitness.

The present study has several limitations. We investigated the *ACTN3* R577X genotype among various sports disciplines only in international-level athletes. However, we did not analyse the genotype based on the sports-specific physiological demand. For example, this study could not distinguish between artistic sports such as artistic gymnasts, rhythmic gymnasts, or figure skating, and ball game sports such as rugby, soccer, badminton, etc. A previous study has reported that ball game sports athletes showed a different R577X genotype distribution between playing positions that exhibits diverse characteristics such as running speed and distance [16]. As our study recruited only international-level sports athletes, we did not analyse their genotypes according to their characteristics. Moreover, the sample size seemed to be small when focusing only on one sport or the position of the athletes. Further studies on larger cohorts are necessary for investigating the relation between physiological demands and gene polymorphism.

## CONCLUSIONS

In conclusion, the *ACTN3* R577X polymorphism was associated with sport categories in international elite athletes in Japan, suggesting the importance of the R carrier genotype for superior performance in sprint/power, martial arts, and ball game sports. In addition, medal-lists in sprint/power sports had a higher frequency of the R allele with the R577X polymorphism.
